# *In vivo* optical imaging of MMP2 immuno protein antibody: tumor uptake is associated with MMP2 activity

**DOI:** 10.1038/srep22198

**Published:** 2016-02-29

**Authors:** Kranthi Marella Panth, Twan van den Beucken, Rianne Biemans, Natasja G. Lieuwes, Marcel Weber, Mario Losen, Ala Yaromina, Ludwig J. Dubois, Philippe Lambin

**Affiliations:** 1 Department of Radiation Oncology (MAASTRO), GROW, MUMC, Maastricht, the Netherlands; 2 Department of Toxicogenomics, GROW, MUMC, Maastricht, the Netherlands; 3 Department of Chemistry and Applied Biosciences, Institute of Pharmaceutical Sciences, ETH Zürich, Zürich, Switzerland; 4 Department of Psychology and Neuropsychology, MHeNS, MUMC, Maastricht, the Netherlands

## Abstract

Matrix metalloproteinase-2 (MMP2) is important in tumorigenesis, angiogenesis and tumor invasion. In this study, we investigated if the Cy5-tagged small immuno protein targeting the catalytic domain of human MMP2 (aMMP2-SIP) detects MMP2 in tumors non-invasively. For this purpose, we generated MMP2 expressing (empty vector EV) and knock-down (KD) HT1080, U373 and U87 cells, which were injected subcutaneously in the lateral flank of NMRI-nu mice. Optical imaging (Optix MX2) performed at 0.5, 2, 4, 8, 24 and 48 hour post injection (h.p.i.) of Cy5 tagged aMMP2-SIP, indicated significantly lower tumor to background ratios at both 24 (P = 0.0090) and 48 h.p.i. (P < 0.0001) for the U87 MMP2-KD compared to control tumors. No differences were found for HT1080 and U373 models. U87 MMP2-KD tumors had significantly lower MMP2 activity (P < 0.0001) than EV tumors as determined by gelatin zymography in tumor sections and lysates, while no differences were observed between EV and MMP2-KD in HT1080 and U373. In line with these data, only U87 MMP2-KD tumors had a reduced tumor growth compared to control tumors (P = 0.0053). aMMP2-SIP uptake correlates with MMP2 activity and might therefore be a potential non-invasive imaging biomarker for the evaluation of MMP2 activity in tumors.

The number of reports on the role of matrix metalloproteinases (MMPs) in cancer progression has tremendously increased over the past years. MMPs play a prominent role in cancer invasion and metastasis to a large degree by disrupting and remodeling of the extracellular matrix (ECM). However, MMPs are also involved in many other important processes during tumorigenesis like proliferation, angiogenesis, apoptosis and migration^1^,^2^,^3^. This raised interest in developing broad-spectrum MMP inhibitors which however failed in subsequent clinical trials because of unspecific targeting related to the extended structure homology of MMPs^4^. Another possible explanation for the failure of the broad-spectrum MMP inhibitors could be that MMPs are thought to be more important in early tumor development, while patients with early stage cancer were not included in these trials^4^. Furthermore, MMPs are tightly regulated at transcriptional level and can have a protective role in tumorigenesis^4^,^5^. Therefore, research efforts are directed towards gaining improved insights on the essential MMPs in tumor progression and to target individual MMPs.

MMP2 (gelatinase A) breaks down type IV collagen, gelatin, elastin, proteoglycans and other collagenous compounds of the ECM^6^. It is upregulated in many cancers^7^ like glioblastomas^8^, melanomas^9^,^10^, breast cancer^11^ and colon cancer^12^. MMP2 plays a vital role in angiogenesis^13^,^14^ and is overexpressed under hypoxic conditions^15^. MMP2 expression in tumors is known to promote invasion and metastasis^16^,^17^ which correlates with the worse prognosis and aggressive behavior associated with these tumors. Moreover, MMP2 inhibition has been shown to cause radiosensitization^18^,^19^, a decrease in tumor growth and invasiveness^13^,^20^,^21^. Altogether this evidence identifies MMP2 as an interesting target for the development of both diagnostic and therapeutic approaches. MMP2 imaging can aid in detecting aggressive tumors, might serve as a surrogate marker of invasion or as biomarker for patient selection in MMP inhibitors trials.

Advances in antibody-based imaging have enabled major progress in detecting and treating cancers^22^,^23^. Antibody-based imaging is sensitive and aids in diagnosis, drug selection, drug development and monitoring treatment efficacy. Imaging strategies using whole IgG antibodies however are limited due to slow antibody clearance from blood^24^,^25^. To circumvent this disadvantage small antibody fragments (minibodies) have been generated by antibody engineering techniques to have superior clearing rates without losing binding characteristics^24^,^25^,^26^. Small chain variable fragments (ScFV) consist of a heavy and a light chain of the variable domains linked by a peptide. ScFv fragments have very fast clearance rates from blood due to small size which is desirable for imaging, but on the other hand, only a small amount of the antibody reaches the tumor^27^. Small immuno protein (SIP) format antibodies have an ScFv fragment linked with the constant domain (ε_S2_CH4) of the human IgE secretory isoform^28^ making them more stable than ScFv fragments. Furthermore, the affinity of SIP is equivalent to full length antibody^28^. Recently an antibody selectively targeting catalytic domain of human MMP2 in small immuno protein (aMMP2-SIP) format has been specifically developed for imaging purposes^29^,^30^.

In this study, we investigated the potential of aMMP2-SIP to detect MMP2 expression in tumors in a non-invasive way. For this, we performed near infrared fluorescence Imaging (NIRF) using Cy5 labeled aMMP2-SIP in mice bearing genetically engineered xenograft tumors. We have evaluated aMMP2-SIP uptake using MMP2 knock-down models, as negative control in different tumor types with varying MMP2 expression and activity.

## Results

### aMMP2-SIP uptake does not solely dependent on MMP2 expression

**Figure 1:**
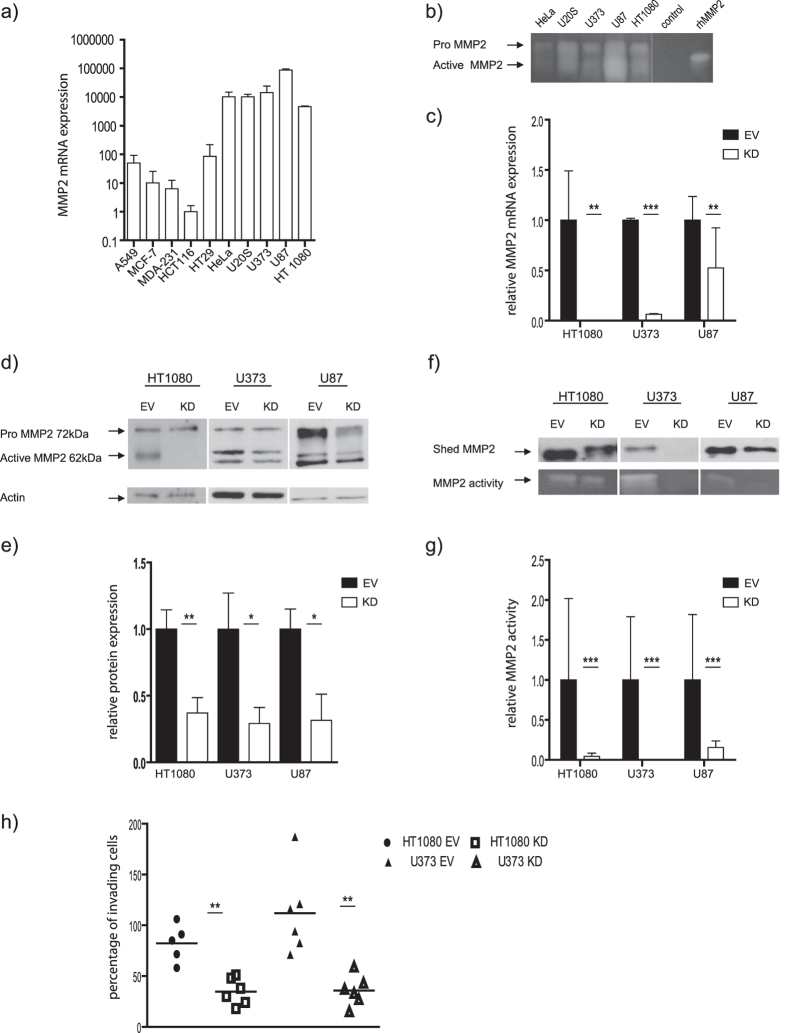
Validation of MMP2-KD models *in vitro*. (**a**) Relative MMP2 mRNA expression and (**b**) MMP2 gelatinase activity in selected cell lines. Recombinant human MMP2 (rhMMP2 66kDa) is taken as positive control and serum free medium as negative control (**c**) Relative MMP2 mRNA expression, (**d**) protein expression and (**e**) active MMP2 protein quantification (**f**) MMP2 gelatinase activity and (**g**) quantification of MMP2 gelatinase activity of empty vector control (EV) and MMP2 knock-down (KD) cells of HT1080, U373 and U87 MMP2-KD compared to control cells. (**h**) Percentage of invading cells in invasive cell lines HT1080 and U373. Data represents the mean +/− SD of at least 3 independent experiments. (***P < 0.0001, **P < 0.01, *P < 0.05).

To select an appropriate model a cell lines panel was first screened for MMP2 mRNA expression by using qPCR (Fig. 1a). Cell lines with high MMP2 expression were further confirmed for MMP2 activity by zymography (Fig. 1b). U87 had the highest MMP2 activity, followed by U20S, HT1080, U373 and Hela. Invasive cell lines HT1080 and U373 with intermediate MMP2 expression and non-invasive U87 with highest MMP2 expression were selected for generation of MMP2-knock-down (KD) models. We silenced MMP2 in HT1080, U373 and in U87 by RNA interference to exclude variation in MMP2 uptake between cell lines that is not attributed to MMP2 expression. The knock-down efficacy was determined at both mRNA and protein levels by using qPCR and western blotting respectively. MMP2 mRNA (Fig. 1c), protein expression (Fig. 1d,e) and activity (Fig. 1f,g) was significantly reduced in all knock-down cell lines compared to empty vector (EV) bearing controls. Both HT1080 and U373 cell lines had a reduced invasion upon silencing MMP2 (Fig. 1h). Altogether this validates the knock-down models *in vitro*.

**Figure 2:**
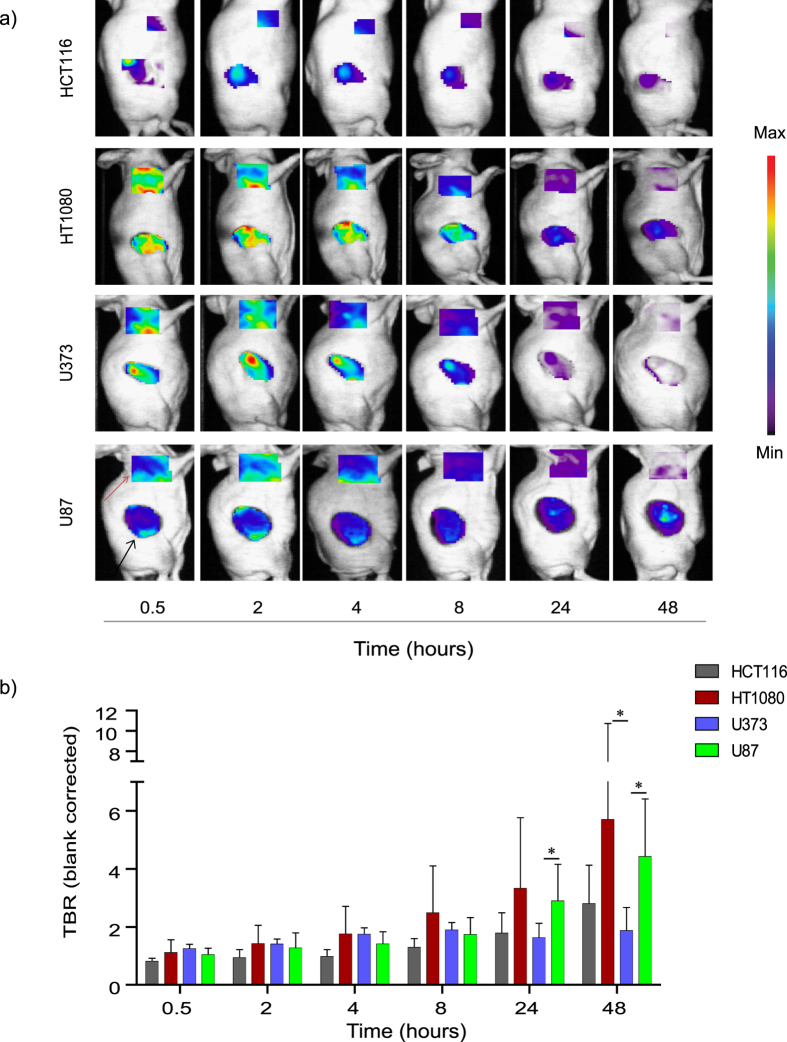
aMMP2-SIP uptake over time in selected models. (**a**) Representative blank (pre-injection) corrected images of HCT116 (n = 5), HT1080 (n = 13), U373 (n = 6) and U87 (n = 8) tumor-bearing mice at 0.5, 2, 4, 8, 24 and 48 hours post injection of tracer. Black arrow indicates tumor, red arrow indicates background. (**b**) Tumor to background ratio quantification over time in different models. (*P < 0.05).

To determine the potential use of aMMP2-SIP as an imaging tracer, control (EV) and MMP2-KD cells of HT1080, U373 and U87 were grown as xenograft tumors in mice. As an additional negative control, HCT116 cells having the lowest MMP2 mRNA expression were grown as xenograft tumors. At an average tumor volume of 294 ± 187 mm^3^, NIRF imaging was performed at several time points after injection of 75 μg of aMMP2-SIP. The optimal time point of imaging was determined by analyzing images acquired at time points from 0.5 to 48 hours post injection (Fig. 2a). Tumor and background fluorescent intensities were high at the early time points. However, the background signal cleared fast while specific signal was retained in the tumors. Tumor to background signal ratio (TBR) started to increase 24 h post injection for most of the tumor models. At 48 h.p.i. the background signal was negligible therefore no further improvement in TBR was expected at later time points. As hypothesized, HCT116 with the lowest MMP2 expression had a low antibody uptake (2.79 ± 1.33). HT1080 with intermediate MMP2 expression had an intermediate uptake but with a large variation within the group (5.69 ± 5.04).Surprisingly, for U373 with intermediate MMP2 expression, TBR was found to be very low (1.86 ± 0.81). U87 with highest MMP2 expression had the highest uptake of all (4.42 ± 1.99) (Fig. 2b).

**Figure 3:**
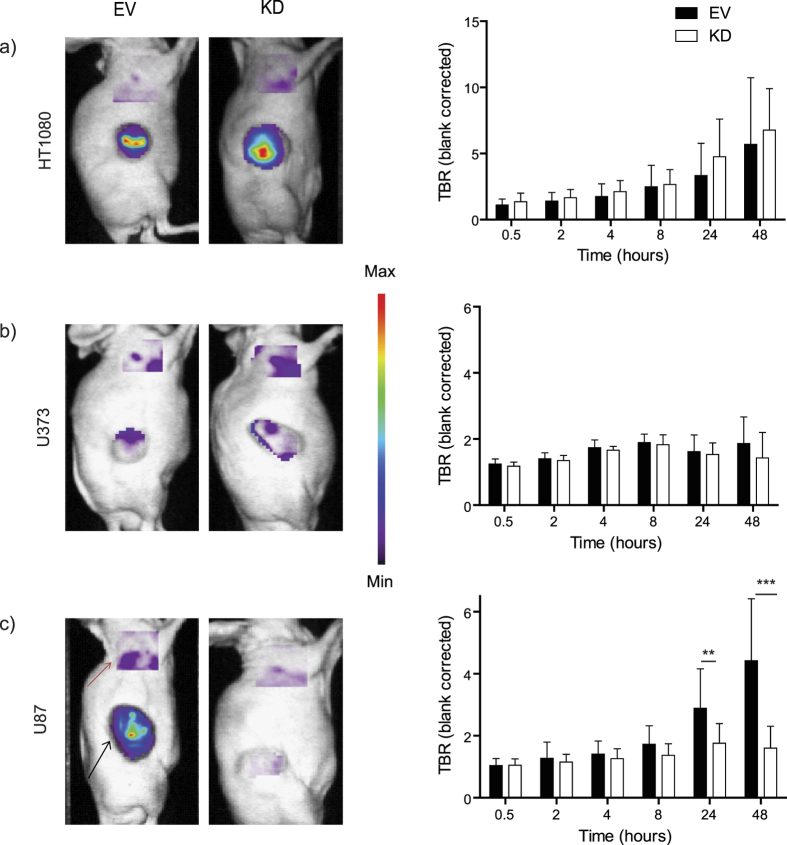
aMMP2-SIP uptake in MMP2 knock-down models compared to control. Representative images at optimal time point 48h.p.i. (Black arrow indicates tumor, red arrow indicates background) and TBR at various time points for (**a**) HT1080 (control n = 13, MMP2-KD n = 16), (**b**) U373 (control n = 6, MMP2-KD n = 6) and (**c**) U87 (control n = 8, MMP2-KD n  =  15) (***P < 0.001, **P < 0.01).

Next, we assessed aMMP2-SIP uptake in the MMP2-KD models. Surprisingly, antibody uptake was not changed in the HT1080 and U373 MMP2-KD tumors (Fig. 3a,b) compared to control. TBR for the U373 models was low (1.86 ± 0.81 for EV and 1.42 ± 0.77 for MMP2-KD) and did not increase with time. Although TBR gradually increased over time in the HT1080 models, no significant (P = 0.49) differences were observed between MMP2-KD (6.76 ± 3.13) and control (5.69 ± 5.04) tumors. On the other hand, the U87 MMP2-KD tumors (1.60 ± 0.70) had a significantly lower aMMP2-SIP uptake (P < 0.0001) compared to the control tumors (4.42 ± 1.99) at optimal time point i.e, 48 h.p.i. (Fig. 3c). Altogether the aMMP2-SIP uptake patterns did not correlate with MMP2 mRNA and protein expression in the xenograft tumors (Supplementary Fig. 1)

### aMMP2-SIP uptake correlates with MMP2 activity and αvβ3 expression

**Figure 4:**
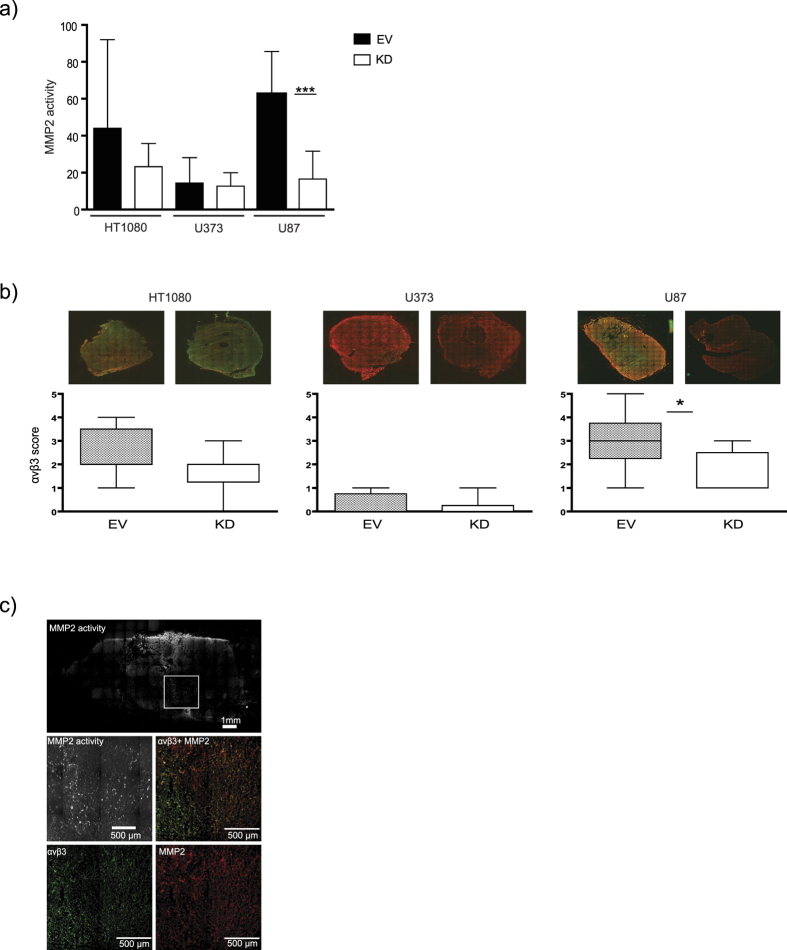
MMP2 activity in tumors. (**a**) MMP2 activity in tumor lysates determined by gelatin zymography. Data represent the mean +/− SD of atleast 5 samples. (**b**) Representative images (green: αvβ3, red: MMP2) and corresponding αvβ3 score for MMP2 knock-down (KD) tumors compared to control (EV) for HT1080, U373 tumors and U87 (*P < 0.05). Data represents median of at least 5 samples (median with 5 and 95 percentile). (**c**) Representative images of *in situ* gelatin zymography on U87 (control) tumor section showing MMP2 activity (Top). Bottom images show regions of high MMP2 activity corresponding with overlapping regions of αvβ3 (green) and MMP2 (red).

**Figure 5:**
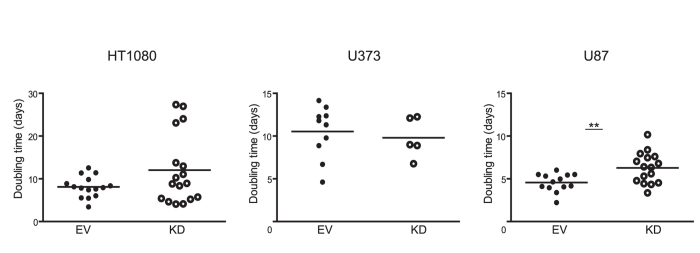
Tumor growth. Tumor doubling time of knock-down (KD) tumors compared to control (EV) for HT1080, U87 and U373. Data represents mean +/− SD (**P < 0.01).

Since aMMP2-SIP did not correlate with MMP2 expression levels, we investigated if aMMP2-SIP uptake could be affected by tumor microenvironmental factors like hypoxia, vasculature or perfused vessels (Supplementary Fig. 2 and supplementary Fig. 3a). Percentage of hypoxic fraction determined by quantification of pimonidazole positive areas in the tumors showed no differences between the control and knockdown tumors in all three tumor models (HT1080 P = 0.9504, U373 P = 0.5366 and U87 P = 0.1577). Percentage of relative vascular area evaluated by CD31 staining also did not significantly differ between the control and knockdown tumors (HT1080 P = 0.2009, U373 P = 0.1069, and U87 P = 0.2927). Next, percentage of perfused fraction in the tumors also did not vary between the control and knockdown tumors (HT1080 P = 0.0554, U373 P = 0.8173 and U87 P = 0.2663). Therefore, hypoxia, vascularization and perfusion did not influence aMMP2-SIP uptake in our models since there were no differences observed between control and MMP2-KD tumors in all three investigated tumor models (Supplementary Fig. 3a). Next we evaluated if difference in MMP2 activity could explain discrepancies in aMMP2-SIP uptake. U87 MMP2-KD xenografts had significantly reduced MMP2 activity compared to controls (P < 0.0001), while no differences were observed in HT1080 and U373 tumors compared to control tumors (Fig. 4a), despite significantly reduced MMP2 levels (Supplementary Fig. 1). In line with the activity data, only MMP2-KD tumors of U87 had a significantly reduced tumor growth rate compared to control tumors (P = 0.0053) (Fig. 5).

Next, we assessed if upstream MMP14 and αvβ3 expression, previously implicated in MMP2 activity regulation^31^,^32^,^33^,^34^, were affected upon silencing MMP2 expression. As expected, no differences were observed between control and MMP2-KD tumors for MMP14 expression (Supplementary Fig. 3b,c). Interestingly, expression of αvβ3 was significantly reduced in MMP2-KD tumors compared to control only in U87 tumors (P = 0.0123) in line with MMP2 activity (Fig. 4b). To find further evidence for this association, we assessed the distribution of active MMP2 and αvβ3 on microregional level in U87 tumor cross sections. Regions of high MMP2 activity were observed in areas with overlapping αvβ3 and MMP2 expression (Fig. 4c). Altogether, the data supports that aMMP2-SIP uptake depends on MMP2 activity which was validated in U87 tumors. In line with this finding, neither MMP2 activity nor aMMP2-SIP uptake was different between EV and KD in the other two tumor models. Data are made publicly available on cancerdata.org (http://dx.doi.org/10.17195/candat.2015.10.6).

## Discussion

MMP2 is considered as a promising druggable target for therapy due to its important role in various processes of tumor progression like angiogenesis, invasion and metastasis. Therefore, imaging active MMP2 can serve as a surrogate marker for aggressive phenotypes. Imaging MMP2 activity has been previously reported^35^,^36^. However, the majority of the studies with activatable probes are difficult to translate into the clinic^37^. Antibody-based imaging has the advantage of selective binding; nonetheless clearance from the blood is slower. In this study we have used an antibody fragment, small immuno protein (SIP) for detecting MMP2 with a faster blood clearance, which thus results in higher TBR. aMMP2-SIP imaging was performed in multiple tumor models with varying MMP2 expression and activity. NIRF imaging of aMMP2-SIP showed that 48 h.p.i. was optimal for imaging with a strong specific signal in the tumor and negligible background. Surprisingly we found that aMMP2-SIP uptake did not exclusively depend on MMP2 expression in our models. Despite of clearly low levels of MMP2 expression in the knock-down models of HT1080 and U373 compared to control, the difference with aMMP2-SIP uptake was not evident; supporting the idea that aMMP2-SIP uptake might not be only due to MMP2 expression. In order to explain the discrepancy in aMMP2-SIP uptake we evaluated tumor microenvironmental parameters such as vasculature, perfusion and hypoxia. However, no major differences were observed that could explain the differences in uptake.

The uptake of the antibody was associated with MMP2 activity in the tumors, suggesting specificity of the antibody towards active MMP2. MMP2 activity in U87 MMP2-KD tumors was significantly lower than the control analogous to the aMMP2-SIP uptake. Interestingly, U87 MMP2-KD tumors with reduced MMP2 activity also had a slower tumor growth rate compared to control tumors. No difference in MMP2 activity was found in both HT1080 and U373 between MMP2-KD and the control tumors. We further determined if MMP14 and integrin αvβ3 which have been previously associated with MMP2 activity^31^,^32^,^33^,^34^, were affected upon silencing MMP2. No differences were observed between control and MMP2KD for both MMP14 and αvβ3 in both HT1080 and U373 tumors. Interestingly, U87 MMP2-KD tumors with low MMP2 activity also had low αvβ3 expression compared to control tumors correlative to the antibody uptake. However, low level of αvβ3 expression in U87 MMP2-KD was unexpected and needs further investigations. It was described that αvβ3 can directly promote activation, maturation^32^ and localization^33^ of MMP2 and that MMP2 was recruited prior to αvβ3 on cell surface before migration and MMP2 was necessary for αvβ3 mediated migration^38^.

Altogether, our study suggests that aMMP2-SIP uptake depends on MMP2 activity. Inhibition of MMP2 appears to be complex due to various mechanisms of activation^39^ and interaction with other molecules such as αvβ3. However if MMP2 activity is detected specifically, it can be beneficial to monitor tumor progression and tumor responses to treatment. In this study, we have demonstrated that aMMP2-SIP uptake was dependent on MMP2 activity in various *in vivo* genetic models. Earlier, our lab successfully used Zirconium-89 (Zr^89^) labeled antibodies for positron emission tomography (PET) imaging^40^. In future, to validate aMMP2-SIP as a potential imaging biomarker by using positron emission tomography (PET), labeling the antibody with Zr^89^ will be performed for feasibility into clinic.

## Conclusions

The optimal time point for imaging with aMMP2-SIP, an antibody targeting the catalytic domain of MMP2, was 48hpi with complete background clearance. The uptake of aMMP2-SIP was not solely dependent on MMP2 expression but rather associated with MMP2 activity. Therefore, aMMP2-SIP can be a potential imaging biomarker for detecting MMP2 activity in tumors.

## Materials and Methods

### Cell lines

A549 (ATCC^®^ CCL-185™), MDA-MB-231(ATCC^®^ HTB-26™), HCT116 (ATCC^®^ CCL-247™), HT-29 (ATCC^®^ HTB-38™), HeLa (ATCC^®^ CCL-2™), U20S (ATCC^®^ HTB-96™), U373 (ATCC^®^ HTB-17), U87 (ATCC^®^ CCL-121™), HT1080 (ATCC^®^ CCL-121™) cells were cultured and maintained in Dulbecco’s modified Eagle medium (DMEM) supplemented with 10% fetal calf serum (FCS). MCF-7 (ATCC^®^ HTB-22™) cells were cultivated in 10% FCS containing RPMI 1640 medium and U373 (ATCC^®^ HTB-17) cells in alpha mem supplemented with 10% FCS and 1 μm/ml L-Glutamine (Westburg).

### Tumor models

We engineered stable knock-down (KD) models of MMP2 in HT1080, U373 and U87 by cloning short hairpin RNAs (shRNAs) specific for MMP2 (TRCN0000051526, target sequence GCAGACATCATGATCAACTTT) into the lentiviral vector pLKO.1 (Sigma). Control cells were made by infecting cells with pLKO.1 without insert (EV). 2 × 10^6^ cells diluted in 50 μl of matrigel were injected subcutaneously in the lateral flank of NMRI-*nu* mice per tumor. Tumor growth was monitored by caliper measurements.

### Real-time PCR Analysis

MMP2 mRNA abundance was measured using MMP2 primers with sequence: 5′-CTTCCAAGTCTGGAGCGATGT-3′ (forward) and 5′-TACCGTCAAAGGGGTATCCAT-3′ (reverse). MMP2 mRNA levels were normalized to the endogenous reference gene 18S ribosomal RNA using the primers 5′-AGTCCCTGCCCTTTGTACACA-3′ (forward) and 5′-GATCCGAGGGCCTCACTAAAC-3′ (reverse).

### Western blotting and Zymography

Protein was isolated from total cell lysates and loaded with Laemmli buffer in 10% SDS-PAGE gels and transferred to nitrocellulose membranes (GE healthcare). Proteins were detected using anti-MMP2 (1:1000) (Biomol), MMP14 (1:1000) (Cedarlane) and anti-actin (1:200000) (MP Biomedicals). Zymography was performed as described before^41^. Cells were incubated overnight in serum-free medium which was collected, concentrated using Amicon ultra centrifugal filter (Ultracel 30kDa) and loaded in the zymogram. For *ex vivo* zymography and western blotting, tumor lysates were prepared by homogenizing tumor samples in radioimmunoprecipitation assay buffer (RIPA buffer) and 20 μg of protein was loaded in gels. Gelatin zymogram (10%), zymogram developing buffer, renaturing buffer and simply blue stain were purchased from Invitrogen. Active MMP2 protein expression and MMP2 activity was quantified by Image J (1.48 v, 64 bit) software.

### Transwell invasion assay

BD Falcon^TM^ cell culture inserts were coated with 1 mg/ml of Matrigel diluted in serum-free medium and incubated at 37 °C overnight. 10^5^ cells suspended in serum-free medium were seeded in the inner chamber and 10% fetal calf serum containing medium was added as chemoattractant in the lower chamber. The invasion system was incubated overnight allowing cell invasion. Cells on the lower side of the insert were stained with 0.5% crystal violet (in 20% methanol) and cells were counted manually.

### aMMP2-SIP antibody production

aMMP2-SIP was produced against the catalytic domain of MMP2 as described before^30^. Monoclonal cells in suspension were cultured in PowerCHO-2 CD medium (Lonza). Purification of the antibody from the culture medium was done by affinity chromatography using protein A Sepharose Fast Flow resin (GE Healthcare) and a protein gel for purity. ELISA and/or SPR (Biacore 3000) were performed for determining functionality before and after the labelling with Cy5. The concentration and labelling ratio was determined using optical density at different wavelengths. aMMP2-SIP was labeled (average of 2–3 labels per molecule) with Cy5 (lumiprobe) dye according to manufacturer’s protocol. The biodistribution of the antibody was described elsewhere^29^,^30^.

### Imaging aMMP2-SIP

Image acquisition and analysis were done as previously described^42^. Near infrared fluorescence (NIRF) imaging was performed using the Optix MX2 (ART, Advanced Research and Technologies) with excitation at 650 nm and emission at 670 nm. 75 μg of aMMP2-SIP was injected intravenously (i.v.) and imaging was performed at various time points i.e. 0.5, 2, 4, 8, 24 and 48 hour post injection of tracer (h.p.i). A blank scan was performed prior to the injection of tracer to enable autofluorescence correction. Delineation of tumor and background was performed using *ART Optix Optiview* software. Regions of interest (ROI) were drawn on the lateral side of upper thorax for obtaining background signal distant from the tumors and excluding regions of clearance organs. ROIs drawn on blank scan for both tumor and background were copied to later time points and therefore were kept constant at all time points. Tumor to background ratios (TBR) were calculated at all time points after correction for auto-fluorescence. After imaging, animals were injected with pimonidazole hypoxia marker [60 mg/kg, intraperitoneal (i.p) Bioconnect] and Hoechst 33342 perfusion marker (15 mg/kg, i.v., Sigma) 1 h and 1 min prior to tumor excision, respectively for histological investigations. The animal experiments were approved by animal ethical committee of Maastricht University and were in accordance with the institutional guidelines for animal welfare.

### Immunohistochemistry

7 μm frozen sections were air dried, acetone fixed and rehydrated by washing with phosphate buffered saline - tween (PBS-Tw-0.2%). Tumor sections were blocked using 5% normal goat serum (NGS) (Vector labs) followed by incubation with primary antibodies diluted in PBS-Tw at 4 °C overnight. After washing with PBS, the sections were incubated with secondary antibodies for 1 h at room temperature. Primary antibodies that were used are rabbit anti-integrin αvβ3 antibody (1:250, Abbiotec), mouse anti-MMP2 (4D3) (1:100, Santa Cruz biotechnology), rabbit anti-pimonidazole (1:250, Bio-connect) and rat anti-mouse CD31 (1:500, BD biosciences). Secondary antibodies were, goat anti-rabbit Alexa 488 for αvβ3 (1:500), goat anti-mouse Alexa 594 for MMP2 (1:500), goat anti-rabbit Alexa 594 for pimonidazole (1:500) and goat anti-rat Alexa 488 for CD31 (1:750) (Invitrogen). Slides were mounted with Shandon™ Immu-Mount™. Photomicrographs were acquired using an Olympus BX51WI fluorescence microscope equipped with a Hamamatsu EM-CCD C9100 digital camera, a motorized stage (Ludl Mac 2000) and a 10x objective. Micromanager 1.4 software was used for automated image acquisition^43^. Stitching of images and quantitative analyses were performed using ImageJ version 1.48 v (http://rsb.info.nih.gov/ij/) by an investigator blinded to the subject coding. Total tumor and necrotic areas were manually delineated. MMP2 and αvβ3 were quantified by visual scoring by two independent investigators (blinded for the treatment conditions) in the total tumor area, where scores from 0 (negative staining) to 5 (high intensity) were used. Percent pimonidazole positive area (hypoxic fraction), relative vascular area and proportion of the perfused vessels were determined in viable tumor compartment as described previously^44^,^45^.

### *In situ* zymography

Tumor sections were air dried for 30 minutes. Dye quenched gelatin (DQ gelatin) (Invitrogen) was prepared in 1% w/v of low melting agarose solution (Sigma) in 1:10 mixture (100 μg/ml) and maintained at 37 °C. 40 μl of this solution was placed directly on top of the section and mounted with a cover slip. The sections were incubated at 37 °C overnight in a humid environment. The sections were then scanned for MMP2 activity and images were stitched as described above.

### Statistics

A nonparametric Mann Whitney test was performed to determine statistical differences in αvβ3 and MMP2 scores between groups. One sample t-test was used to test whether average change in MMP2 activity is significantly different from 1. For all other statistical analyses between two groups the unpaired t test was performed. All statistics and graphs were made in Graphpad Prism (v5.03). A two- sided P-value smaller than 0.05 was considered statistically significant.

## Additional Information

**How to cite this article**: Panth, K. M. *et al. In vivo* optical imaging of MMP2 immuno protein antibody: tumor uptake is associated with MMP2 activity. *Sci. Rep.* **6**, 22198; doi: 10.1038/srep22198 (2016).

## Supplementary Material

Supplementary Information
