# Supplementary material for: In vivo optical imaging of MMP2 immuno protein antibody: tumor uptake is associated with MMP2 activity
Source: Sci Rep. 2016 Feb 29;6:22198. doi: 10.1038/srep22198 (PMC4770595; doi:10.1038/srep22198)
Supplement: Supplementary Information [file srep22198-s1.pdf]

In vivo optical imaging of MMP2 immuno protein antibody: tumor uptake is associated with MMP2 activity

Kranthi Marella Panth[1*], Twan van den Beucken[1,2], Rianne Biemans[1], Natasja G Lieuwes[1], Marcel Weber[3], Mario Losen[4], Ala Yaromina[1], Ludwig J Dubois[1#], Philippe Lambin[1#]

[1]Department of Radiation Oncology (MAASTRO), GROW, MUMC, Maastricht, the Netherlands

[2]Department of Toxicogenomics, GROW, MUMC, Maastricht, the Netherlands

[3]Department of Chemistry and Applied Biosciences, Institute of Pharmaceutical Sciences, ETH Zürich, Zürich, Switzerland

[4]Department of Psychology and Neuropsychology, MHeNS, MUMC, Maastricht, the Netherlands

[#] indicates equal contribution

Running title: Imaging MMP2 activity with small immuno protein antibody

Key words: MMP2 activity, matrix metalloproteinase, preclinical imaging, integrin alpha v beta 3

Number of pages: 22

Number of figures: 5

Number of supplementary figures: 3

* Corresponding author: kranthi.panth@maastrichtuniversity.nl

Department of Radiation Oncology (Maastro Lab), Grow - School for Oncology and Developmental Biology

Maastricht University Medical Centre, Universiteitssingel 50/23, PO Box 616, 6200 MD Maastricht

The Netherlands

Phone: +31 43 388 1586/2453

Supplementary information:

Supplementary figure 1: *MMP2 expression in tumors*

MMP2 mRNA expression determined by qPCR (mean +/- SD) and MMP2 score (median with 5 and 95 percentile of atleast 5 samples) determined by immunohistochemistry in HT1080, U373 and U87 control (EV) and MMP2-KD (KD) tumors (***P< 0.0001, ** P< 0.01, *P <0.05).

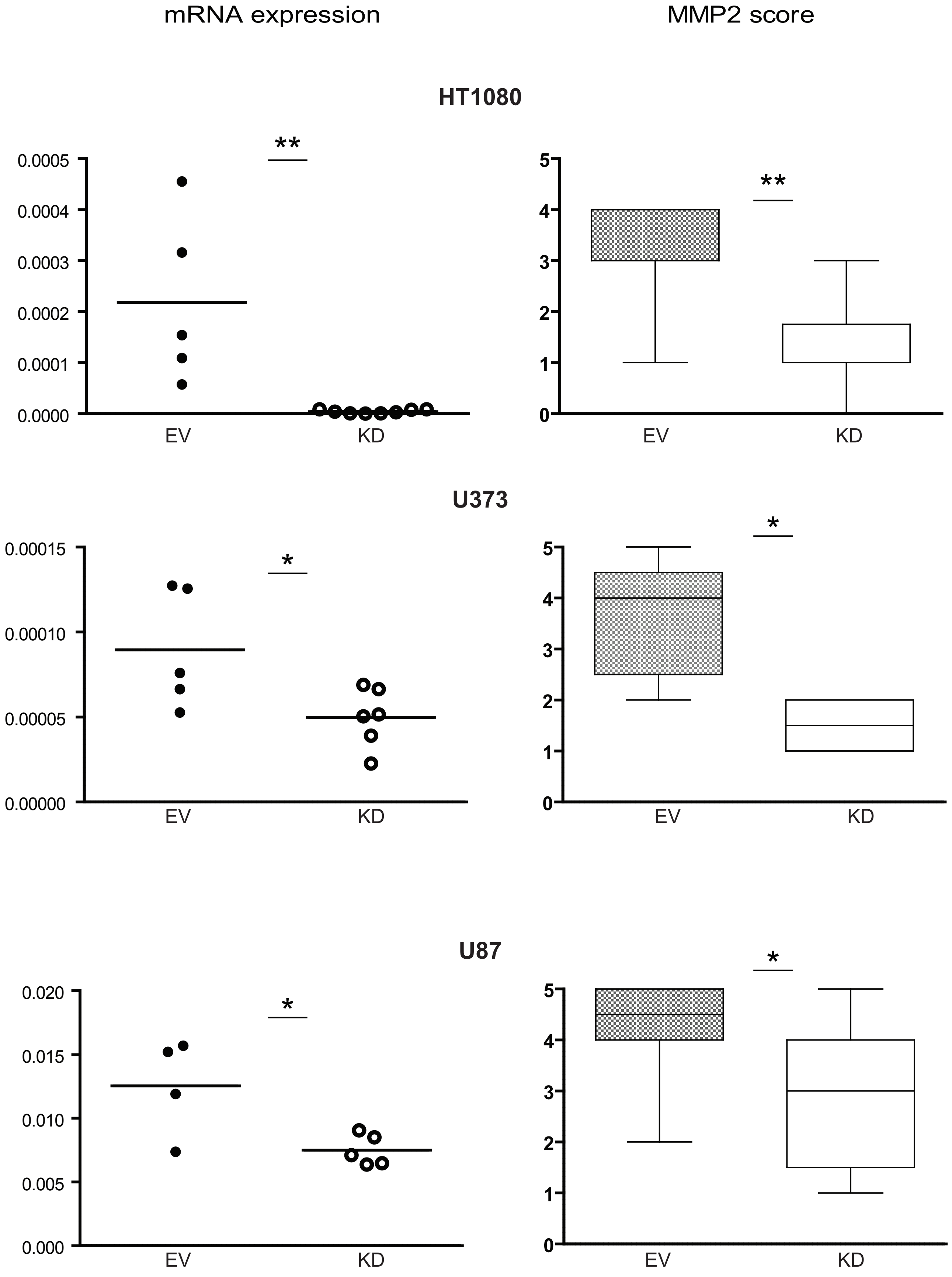

mRNA expression
MMP2 score
HT1080
**
**
0.0005
0.0004
0.0003
0.0002
0.0001
0.0000
EV
KD
5
4
3
2
1
0
EV
KD
U373
*
*
0.00015
0.00010
0.00005
0.00000
EV
KD
5
4
3
2
1
0
EV
KD
U87
*
*
0.020
0.015
0.010
0.005
0.000
EV
KD
5
4
3
2
1
0
EV
KD

Supplementary figure 2: *Representative images for tumor microenvironment*

Immunohistochemistry images of HT1080, U373 and U87 control (EV) and MMP2-KD (KD) tumors stained for Hypoxia [pimonidazole (pimo)] in green, blood vessels (CD31) in red and perfused vessels (Hoechst) in blue. White bar represents a scale of 1mm.

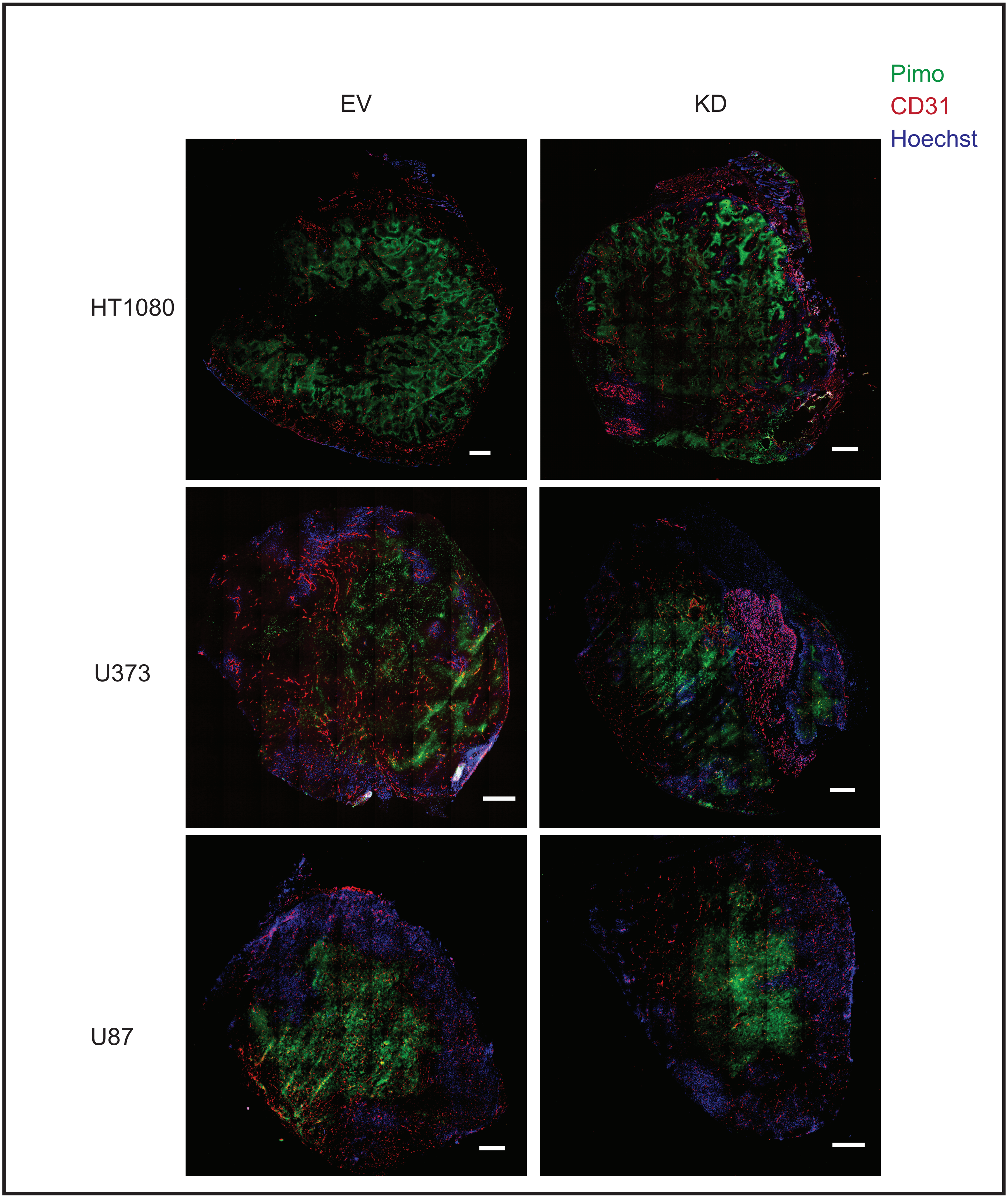
EV
KD
Pimo
CD31
Hoechst
HT1080
U373
U87

Supplementary figure 3: *Tumor microenvironmental characteristics and MMP14 expression*

(a) Quantification of the hypoxic fraction, microvessel density and the percent perfused vessels in HT1080 U373 and U87 control (EV) and MMP2-KD (KD) tumors. (b) Western blot analysis and (c) quantification of active MMP14 expression in tumor lysates. Data represents mean +/- SD of at least 5 samples.

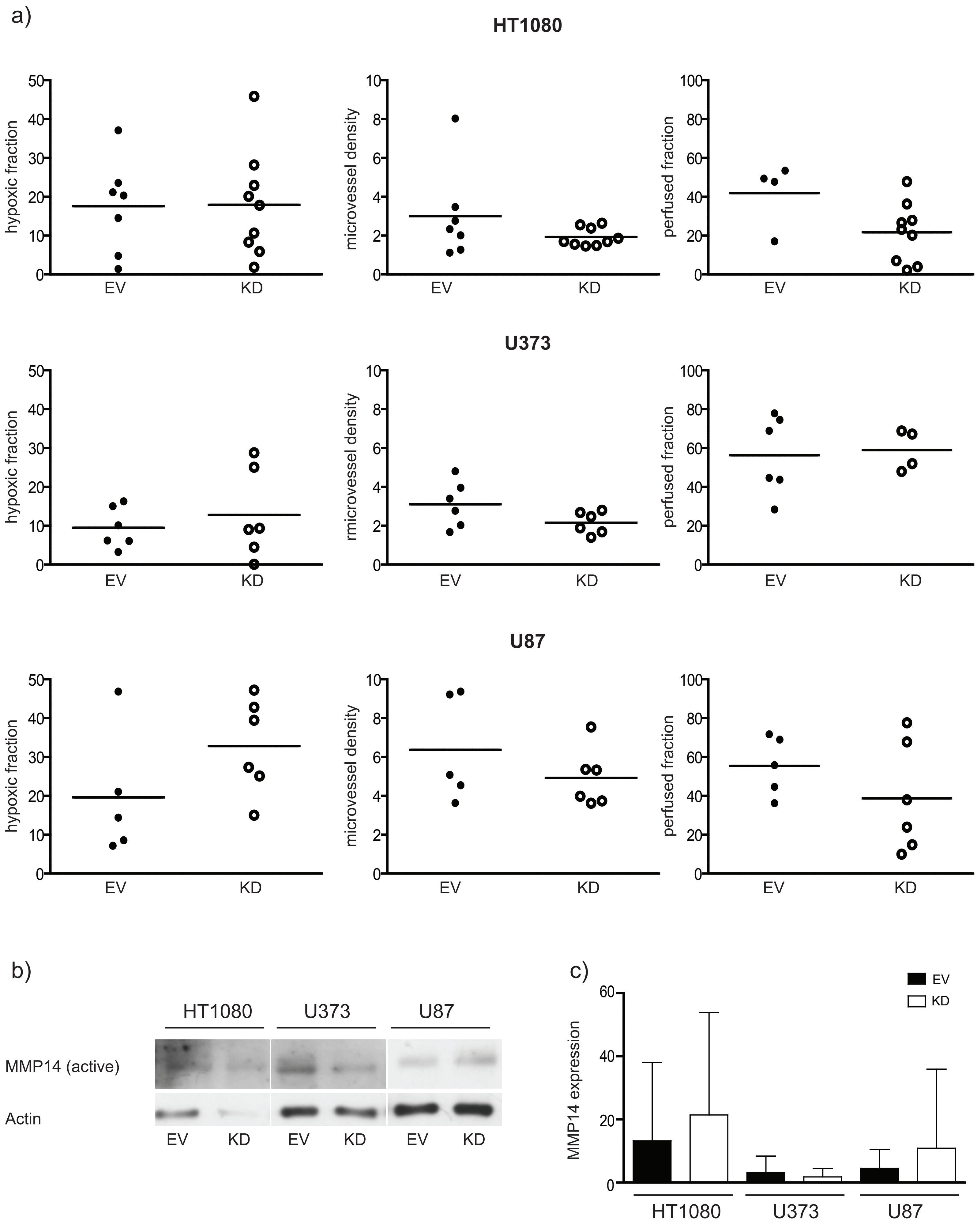
a)
HT1080
hypoxic fraction
microvessel density
perfused fraction
EV
KD
U373
rmicrovessel density
U87
b)
HT1080
U373
U87
MMP14 (active)
Actin
EV
KD
c)
MMP14 expression
EV
KD
HT1080
U373
U87
